# Supplementary material for: Numerical Model and Experimental Validation for Laser Sinterable Semi-Crystalline Polymer: Shrinkage and Warping
Source: Polymers (Basel). 2020 Jun 18;12(6):1373. doi: 10.3390/polym12061373 (PMC7361694; doi:10.3390/polym12061373)
Supplement: Supplementary file 1 [file polymers-12-01373-s001.pdf]

polymers

MDPI

Supplementary Materials

Numerical model and experimental validation for laser sinterable semi-crystalline polymer: shrinkage and warping

Jiang Li [1], Shangqin Yuan [2,3,*], Jihong Zhu [1,2,*], Shaoying Li [1], and Weihong Zhang [1]

[1] State IJR Center of Aerospace Design and Additive Manufacturing, School of Mechanical Engineering, Northwestern Polytechnical University, 710072 Xi'an, Shaanxi, China; jh.zhu@nwpu.edu.cn
[2] MIIT Lab of Metal Additive Manufacturing and Innovative Design, NPU-QMUL Joint Research Institute, Northwestern Polytechnical University, 710072 Xi'an, Shaanxi, China; shangqin.yuan@nwpu.edu.cn
[3] Unmanned System Research Institute, Northwestern Polytechnical University, 710072 Xi'an, Shaanxi, China; shangqin.yuan@nwpu.edu.cn
* Correspondence: shangqin.yuan@nwpu.edu.cn; jh.zhu@nwpu.edu.cn；
Tel.: +86-150-9115-6319；+86-137-2045-5640

Received: date; Accepted: date; Published: date

1. The computing method of $t_{1/2}$ in kinetics theory

The time $t_{1/2}$ indicating the half amount of polymer chains being crystallized is given by

$$t_{1/2}(c) = c_i + \left[t_{1/2}(i+1) - t_{1/2}(i)\right] / \left[c(i+1) - c(i)\right] \cdot \left[c - c(i)\right] \tag{S1}$$

where *i* is the index of the column such that *c* is comprised between the *ith* and *ith+1* column in Table S1 [1]. For example, in a case where *c=1.2*, then *i=3*. For cooling rates beyond the range of 0.1 to 3.2, the extremum on the same side was used. For example, if c=5.0, then $t_{1/2}$=5.5.

Table S1. Half crystallization times of PA12 corresponding to cooling rate

Cooling rate (°C /min)	0.1	0.2	0.4	0.8	1.6	3.2
$t_{1/2}$ (min)	66.9	38.4	24.4	14.7	9.1	5.5
i	0	1	2	3	4	5

2. The orthogonal array used in this experiment.

Table S2. Taguchi L_{16} (4^5) orthogonal array

Experiment No.	Levels of each parameter				
	P	*v*	*h*	*t*	*θ*
1	1	1	1	1	1
2	1	2	2	2	2
3	1	3	3	3	3
4	1	4	4	4	4
5	2	1	2	3	4
6	2	2	1	4	3
7	2	3	4	1	2
8	2	4	3	2	1
9	3	1	3	4	2
10	3	2	4	3	1

11	3	3	1	2	4
12	3	4	2	1	3
13	4	1	4	2	3
14	4	2	3	1	4
15	4	3	2	4	1
16	4	4	1	3	2

3. The parameter cases for calculating melting pool size and temperature

Table S3. Different parameters used for calculating melting pool size and temperature

No.	P(W)	v(mm/s)	h(mm)	t(mm)	Energy density (J/mm^3)
1	7	4500	0.25	0.1	0.0622
2	10	4500	0.25	0.1	0.0889
3	15	4500	0.25	0.1	0.1333
4	20	4500	0.25	0.1	0.1778
5	25	4500	0.25	0.1	0.2222
6	30	4500	0.25	0.1	0.2667
7	35	4500	0.25	0.1	0.3111
8	20	3000	0.25	0.1	0.2667
9	20	3500	0.25	0.1	0.2286
10	20	4000	0.25	0.1	0.2000
11	20	4500	0.25	0.1	0.1778
12	20	4500	0.15	0.1	0.2963
13	20	4500	0.2	0.1	0.2222
14	20	4500	0.25	0.1	0.1778
15	20	4500	0.3	0.1	0.1481

4. The shrinkage and warping of orthogonal experiment and modeling

Table S4. Shrinkage of orthogonal designed experiment and modeling

Experiment No.	Experiment (%)			Modeling (%)
	$L\varepsilon_1$	$L\varepsilon_2$	$L\varepsilon_3$	
1	4.1192	4.0008	4.0216	3.3128
2	4.5304	4.5616	4.5280	3.4224
3	3.7760	3.8672	3.7520	2.8997
4	3.5360	3.3880	3.4800	2.5530
5	4.9152	4.7896	4.8848	3.7154
6	4.5704	4.6008	4.6768	3.4355
7	4.4760	4.5672	4.5264	3.2828
8	4.0224	3.9192	3.9720	3.1685
9	2.8472	2.7624	2.9056	2.6163
10	4.2312	4.1888	4.2784	3.3258
11	4.7120	4.8096	4.8472	3.5676
12	4.8848	4.9104	4.8568	3.6150
13	4.8736	4.9096	4.9888	3.7845
14	3.9528	3.9616	4.0352	3.1917
15	4.4192	4.4776	4.5120	3.3628
16	4.3064	4.3480	4.4856	3.7507

Table S5. Warping of orthogonal designed experiment and modeling

Experiment No.	Experiment(mm)						Modeling(mm)
	u_1	u_2	u_3	u_4	u_5	u_6	

1	0.5063	0.5199	0.4903	0.4894	0.5100	0.5201	0.4806
2	0.2817	0.2765	0.2823	0.3011	0.3026	0.3096	0.2830
3	0.3235	0.3178	0.2990	0.2885	0.3064	0.2966	0.2346
4	0.1833	0.1771	0.1743	0.1659	0.1524	0.1490	0.0121
5	0.3162	0.3166	0.3427	0.3259	0.3430	0.3476	0.3449
6	0.3785	0.3810	0.3767	0.3597	0.3584	0.3495	0.3323
7	0.2027	0.2158	0.2036	0.2220	0.2071	0.2238	0.2494
8	0.2865	0.2797	0.304	0.2808	0.3013	0.2745	0.2619
9	0.2360	0.2241	0.2324	0.2145	0.2140	0.2080	0.0113
10	0.2418	0.2612	0.2615	0.2654	0.2708	0.2671	0.2803
11	0.4279	0.4543	0.4279	0.4450	0.4227	0.4772	0.4707
12	0.3466	0.3560	0.3602	0.3629	0.3460	0.3271	0.3882
13	0.4174	0.4236	0.4429	0.4416	0.4200	0.4063	0.3832
14	0.4319	0.4573	0.4379	0.4001	0.4315	0.4333	0.4611
15	0.3949	0.4057	0.3906	0.4162	0.4079	0.4267	0.3157
16	0.4060	0.4137	0.4019	0.4221	0.4165	0.4358	0.3640

5. The ANOVA results of shrinkage and warping results

Table S6. ANOVA of shrinkage-experiment

Factor	Sum of square	DOF	Mean square	F	Sig.
P	0.034	3	0.011	176	***
v	0.008	3	0.003	48	***
h	0.112	3	0.037	592	***
t	0.051	3	0.017	272	***
θ	0.024	3	0.008	128	***
Error	0.002	32	6.25E-5		

*. Significance level

Table S7. ANOVA of warping-experiment

Factor	Sum of square	DOF	Mean square	F	Sig.
P	0.215	3	0.072	384	***
v	0.053	3	0.018	96	***
h	0.357	3	0.119	634.667	***
t	0.102	3	0.034	181.333	***
θ	0.098	3	0.033	176	***
Error	0.015	80	1.875E-4		

*. Significance level

6. Reference

1. VASTOLA, G.; BAI, J.; YUAN, S. APPARATU AND METHOD TO PREDETERMINE GEOMETRICAL CHANGES OF AN OBJECT AND OBJECT BUILT BY ADDITIVE MANUFACTURING. 2018.
